# Whole-body MRI including diffusion-weighted MRI compared with 5-HTP PET/CT in the detection of neuroendocrine tumors

**DOI:** 10.1080/03009734.2016.1248803

**Published:** 2016-11-29

**Authors:** Lina Carlbom, José Caballero-Corbalán, Dan Granberg, Jens Sörensen, Barbro Eriksson, Håkan Ahlström

**Affiliations:** a Institute of Surgical Sciences, Department of Radiology, Uppsala University, Uppsala, Sweden;; b Department of Medical Sciences. Uppsala University, Uppsala, Sweden

**Keywords:** Diffusion-weighted MRI, magnetic resonance imaging, neuroendocrine tumors, positron-emission tomography, whole-body imaging

## Abstract

**Aim:**

We wanted to explore if whole-body magnetic resonance imaging (MRI) including diffusion-weighted (DW) and liver-specific contrast agent-enhanced imaging could be valuable in lesion detection of neuroendocrine tumors (NET). [11C]-5-Hydroxytryptophan positron emission tomography/computed tomography (5-HTP PET/CT) was used for comparison.

**Materials and methods:**

Twenty-one patients with NET were investigated with whole-body MRI, including DW imaging (DWI) and contrast-enhanced imaging of the liver, and whole-body 5-HTP PET/CT. Seven additional patients underwent upper abdomen MRI including DWI, liver-specific contrast agent-enhanced imaging, and 5-HTP PET/CT.

**Results:**

There was a patient-based concordance of 61% and a lesion-based concordance of 53% between the modalities. MRI showed good concordance with PET in detecting bone metastases but was less sensitive in detecting metastases in mediastinal lymph nodes. MRI detected more liver metastases than 5-HTP PET/CT.

**Conclusion:**

Whole-body MRI with DWI did not detect all NET lesions found with whole-body 5-HTP PET/CT. Our findings indicate that MRI of the liver including liver-specific contrast agent-enhanced imaging and DWI could be a useful complement to whole-body 5-HTP PET/CT.

## Introduction

Tumors arising from neuroendocrine cells are a heterogeneous family of malignancies that are typically slow growing and well differentiated. Metastatic disease followed by hormonal symptoms is often (but not always) found at the time of diagnosis. Small intestinal neuroendocrine tumors (Si-NET) and pancreatic neuroendocrine tumors (P-NET) are frequently diagnosed with liver metastases, whereas lung carcinoids (LCC), especially atypical, primarily have intrathoracic lymph node metastases at diagnosis and later develop distant metastases in the liver, bone, brain, and subcutaneous tissues (1,2). As widespread disease leads to poor prognosis, correct visualization of disease extension is of importance for choice of treatment in order to improve survival.

5-Hydroxytryptophan (5-HTP) is a precursor in serotonin synthesis and is taken up and accumulated in neuroendocrine tumors (NET). Whole-body positron emission tomography (PET) imaging with ^11^C-labeled 5-HTP can be used as a universal imaging technique to visualize malignant lesions in a range of well-differentiated NET (3). Diffusion-weighted imaging (DWI) provides functional information based on the random motion of water molecules in intra- and extracellular spaces (4). DWI has become an increasingly popular method for lesion detection and characterization in oncologic imaging (5). Tumors with high cellular density have an impeded diffusion (6). NET consists of tightly packed cells, thus making DWI an appealing method for detection of the lesions. Since patients with NET have a high long-term survival rate, with repeated follow-up examinations, and are often relatively young, a radiation-free technique for lesion detection would be of value. Whole-body imaging, covering at least neck to groin, with DWI maximum intensity projections (MIP) of the whole area, provides an overview and is advantageous since NET can metastasize to many different locations. Only a few studies assessing the possible benefit of whole-body DWI in the detection of NET have been performed (7,8); further studies in this area are warranted.

The aim of this exploratory study was to evaluate the possible benefit of whole-body magnetic resonance imaging (MRI) including DWI, covering base of the skull to upper thigh, and liver-specific contrast agent-enhanced imaging in lesion detection of NET. 5-HTP PET/computed tomography (CT), a method with proven high sensitivity and specificity in NET lesion detection (3), was used for comparison.

## Materials and methods

### Patients

Patient characteristics have been summarized in Table 1. The inclusion criteria consisted of patients with histopathologically diagnosed well-differentiated NET (Ki 67 equal to or less than 20%) and suspicion of recurrent disease based on elevated tumor markers, either general neuroendocrine tumor markers (such as chromogranin A or pancreas polypeptide) or syndrome-specific hormonal marker (e.g. insulin, 5-hydroxyindoleacetic acid, cortisol), where somatostatin receptor scintigraphy and contrast-enhanced CT yielded unclear findings and 5-HTP PET/CT had been performed to obtain more information in order to enable appropriate therapeutic decisions. Twenty-one patients who underwent whole-body 5-HTP PET/CT and whole-body MRI including DWI within 3 months (11 females and 10 males, age 19–71 years) were prospectively included. In order to investigate whether MRI can provide an alternative to 5-HTP PET/CT in detection of NET metastases in the liver, seven additional patients (5 females and 2 males, age 32–74 years) who had undergone upper abdomen MRI including DWI (similar liver protocol as whole-body MRI) and whole-body 5-HTP PET/CT within 3 months were retrospectively included. Since well-differentiated NET are slow growing tumors the time limit between the two different studies was set at 3 months; however, the median time between MRI and PET examinations was only 1 day. The patients underwent imaging during the period May 2008 to February 2013.

**Table 1.** Patients characteristics.

	All(*n* = 28)	Whole-body(*n* = 21)	Upperabdominal(*n* = 7)
Females	16 (57%)	11 (52%)	5 (71%)
Age at diagnosis (years)^a^	49 (19–74)	47 (19–71)	61 (32–74)
Time between MR and PET (days)^a^	1 (0–78)	1 (0–78)	36 (1–49)
Time diagnosis to MRI (months)^a^	41 (2–299)	38 (2–299)	50 (3–158)
Histopathology			
Si-NET	4 (14%)	4 (19%)	0
P-NET	13 (46%)	8 (38%)	5 (72%)
LCC	8 (29%)	7 (33%)	1 (14%)
Thymic NET	1 (4%)	1 (5%)	0
Ovarian NET	1 (4%)	0	1 (14%)
Unknown origin	1 (4%)	1 (5%)	0
Primary tumor resected	25 (89%)	18 (86%)	7 (100%)
Elevated chromogranin A	12 (43%)	7 (33%)	5 (72%)
Ki 67 (%)^a^	5 (1–20)	5 (1–20)	7 (1–16)

a Median (minimum–maximum).

LCC: lung carcinoid; P-NET: pancreatic neuroendocrine tumor; Si-NET: small intestine neuroendocrine tumor.

Three patients had functioning NET (2 LCC with ectopic Cushing and 1 P-NET with insulinoma syndrome). The remaining patients had non-functioning tumors (6 LCC, 12 EPT, 4 Si-NET, 1 thymic NET, 1 ovarian NET, and 1 NET of unknown origin). Seven patients had ongoing anti-tumoral treatment at the time of imaging (3 somatostatin analog, 3 chemotherapy, 1 combined somatostatin analog and mTOR inhibitor).

The study was approved by the local ethics committee.

### Imaging

#### 5-HTP PET/CT

5-HTP was synthesized in a multienzymatic reaction according to previously described procedures (9). Patients were fasted for at least 4 h prior to PET. Carbidopa was given in a dose of 200 mg 1 h before PET to improve image quality by reducing tracer decarboxylation (10). A dose of 5 MBq/kg body weight 5-HTP was injected intravenously in an antecubital vein 20 min prior to PET acquisition. PET/CT was performed in a GE Discovery ST16 (GE Healthcare, Waukesha, ML) hybrid scanner with a low-dose CT for attenuation correction (140 kV, auto-mA 20–80 mA) and PET with 3 min per 15.7 cm bed position, covering regions from the symphysis to the neck, typically obtained in five bed positions. The typical total radiation dose of the examination was 7 mSv, and the typical total examination time was 20 min. PET images were corrected for attenuation, scatter, and decay, and reconstructed to a 50 cm axial field of view in a 128 × 128 matrix using a built-in iterative reconstruction algorithm with 2 iterations and 21 subsets.

#### Whole-body MRI

In seven patients examinations were performed with a 1.5 T MR scanner (Gyroscan Intera, Philips, Best, The Netherlands). The whole-body built-in transmit-receiver coil and four acquisition stations were used, covering head to upper thigh. Whole-body imaging was done using coronal T1-weighted spin echo (T1W) with breath-holding in abdomen and thorax, and using coronal fat-suppressed T2-weighted STIR (T2W-STIR) with respiratory triggering in thorax and abdomen. Whole-body DWI with background body signal suppression was performed in transversal planes. DWI acquisitions were performed during free breathing. Whole-body MIP images were then reconstructed. In addition, separate transversal imaging of the upper abdomen using a surface coil was performed, consisting of T2-weighted turbo spin echo (T2W-TSE) with respiratory triggering, T1-weighted breath-hold in-and-out-phase gradient echo (T1W-GE), and dynamic series of T1-weighted 3D gradient echo (THRIVE) in upper abdomen before and after administration of contrast agent, Gd-DTPA (Magnevist, Bayer) 0.2 mL/kg. Dynamic phases after contrast agent administration were monitored, including arterial phase, portal venous phase, and equilibrium phase. Total examination time was approximately 45 min (main imaging parameters in Table 2).

**Table 2.** Whole-body MRI, Philips.

	T1 cor	T2 STIR cor	T1 transversal	T2 transversal	THRIVE	DWI
Sequence type	TSE	TSE	In and out of phase	TSE	T1 3D-GRE	EPI
TR/TE/TI (ms)	326/18/0	1666/64/165	178/2.3 and 4.6/0	1666/100/0	3.6/1.72/0	3222/70/180
Thickness/gap (mm)	6/1	6/1	8/0.8	8/0.8	4/–2	6/0
Voxel size (mm)	0.95 × 0.95 × 6.0	0.95 × 0.95 × 6.0	1.56 × 1.55 × 8.0	0.78 × 0.78 × 8.0	1.56 × 1.57 × 2.0	1.51 × 1.50 × 6.0
Matrix	368 × 140	320 × 135	192 × 106	400 × 246	192 × 136	112 × 75
Bandwidth (Hz/pixel)	449	504	522	219	543	22
Signal averages (*n*)	2	2	2	2	2	2
B values (s/mm^2^)						0 and 1000
Slices/station (*n*)	33–45	33–45	25–30	25–30	100–105	33–45

TE: echo time; TI: inversion time; TR: repetition time.

In 14 patients examinations were performed with a 1.5 T MR scanner (Magnetom Avanto, Siemens, Germany). The imaging was done using continuously moving table acquisition and seven surface coils covering base of the skull to upper thigh. Whole-body DWI was performed with seven acquisition stations and background body signal suppression in transversal planes. DWI acquisitions were performed during free breathing. Whole-body three-dimensional MIP images were then reconstructed. In addition, whole-body transversal T2-weighted BLADE, transversal T1-weighted breath-hold in-and-out-phase gradient echo imaging of the abdomen and thorax, and transversal T1-weighted imaging of the neck were performed. Transversal dynamic series of T1-weighted 3D gradient echo (VIBE) in the upper abdomen before and after administration of liver-specific contrast agent Gd-EOB-DTPA (Primovist, Bayer) 0.1 mL/kg were performed. Dynamic phases after administration of contrast agent included arterial phase, portal venous phase, and equilibrium phase. A hepato-biliary late phase was also performed. Total examination time was approximately 45 min (main imaging parameters in Table 3).

**Table 3.** Whole-body MRI, Siemens.

	T1 thorax and abdomen	T1 neck	T2 thorax and abdomen	T2 neck	VIBE	DWI
Sequence type	In and out of phase	FSE	BLADE	BLADE	T1 3D-GRE	EPI
TR/TE/TI (ms)	150/2.38 and 4.76/0	400/9.1/0	6140/116/0	4400/99/0	3.44/1.24/0	8200/78/160
DF (%)	20	10	20	20	20	0
Voxel size (mm)	2.6 × 1.8 × 5.0	1.1 × 0.8 × 5.0	1.8 × 1.8 × 5.0	1.0 × 1.0 × 5.0	2.1 × 1.5 × 3.5	2.9 × 2.9 × 5.0
Matrix	256 × 179	384 × 288	256 × 233	320 × 299	256 × 179	160 × 160
Bandwidth (Hz/pixel)	480	130	362	363	430	1644
Signal averages (*n*)	1	1	1	1	1	2
B values (s/mm^2^)						50 and 1000
Slices/station (*n*)						30

DF: distortion factor; TE: echo time; TI: inversion time; TR: repetition time.

#### Upper abdomen MRI

In seven patients MRI of the upper abdomen was performed using 1.5 T MR scanner (Gyroscan Intera, Philips, Best, The Netherlands) and a similar upper abdominal protocol as the one included in the whole-body protocol for the Gyroscan Intera scanner described above. A coronal fat saturated T2-TSE (SPAIR) and transversal DWI with low B values of 0 and 50 and high B values of 700 or 800 were added to the protocol. In four of these patients liver-specific contrast agent Gd-EOB-DTPA (Primovist, Bayer) 0.1 mL/kg was used, and in three patients Gd-BOPTA (Multihance, Bracco) 0.1 mL/kg. Dynamic series before and after administration of contrast agent and a late phase were obtained. Total examination time was approximately 35 min, excluding the prolonged waiting time between the other sequences and the late phase sequence required in patients examined with Gd-BOPTA. Main imaging parameters are shown in Table 4.

**Table 4.** Upper abdomen MRI.

	T2 SPAIR cor	T1 transversal	T2 transversal	THRIVE	DWI
Sequence type	TSE	In and out of phase	TSE	T1 3D-GRE	EPI
TR/TE (ms)	621/80	182/2.3 and 4.6	1696/90	3.9/1.83	1580/63
Thickness/gap (mm)	5/1	5/0.5	8/0.8	4.0/–2	8/0.8
Voxel size (mm)	0.84 × 0.84 × 5.0	1.56 × 1.55 × 5.0	0.73/0.73/8.0	0.98 × 0.97 × 2.0	1.46 × 1.48 × 8.0
Matrix	312 × 314	192 × 114	376 × 252	188 × 168	124 × 87
Bandwidth (Hz/pixel)	391	522	261	434	42.3
Signal averages (*n*)	2	1	2	1	4
B values (s/mm^2^)					0, 50, and 700 or 800
Slices/station (*n*)	35	40	30	110	30

TE: echo time; TR: repetition time.

### Imaging evaluation

The evaluation of the MRI images was carried out individually by two radiologists, one of whom was a senior consultant. The readers were blinded to the result of the PET/CT. When interpretation differed between the readers, the images were re-evaluated and a consensus was reached. PET/CT examinations were evaluated by a specialist in nuclear medicine, performing the clinical report.

#### 5-HTP PET/CT

A lesion was considered positive when there was a greater focal 5-HTP uptake than background activity incompatible with normal anatomy/physiology. The location of the 5-HTP uptake was verified by CT.

#### MRI

The MIP images of whole-body DWI and transversal DWI sequences were assessed using a B value of 1,000 mm^2^/s. Apparent diffusion coefficient (ADC) maps were used to rule out a T2-shine-through effect (11) and B value 0 images to get anatomical information (12). A lesion was defined as a focal area ≥1 cm in diameter with equal or higher signal intensity than the signal intensity from the organ with highest signal intensity in each station, i.e. in the neck region it was compared to the brain, and in other regions to the bone marrow (13). The DWI images (the original transversal images and the MIP reconstructions) were assessed together with the conventional T1 and T2 sequences. A positive DWI finding was confirmed on the conventional T1- and T2-weighted images to rule out probable artifacts in whole-body DWI.

Liver contrast-enhanced images and DW images were assessed for liver metastases. A liver lesion was considered a metastasis if it showed hypervascularity with contrast enhancement on arterial phase images and washout, resulting in an iso- or hypointense signal in late dynamic and hypointense signal in hepato-biliary phase, or if it showed a signal intensity pattern indicating washout combined with high signal intensity on the DWI sequence, even if the size was <1 cm.

Conventional MR images were assessed for morphological findings compatible with tumor lesions.

#### Lesion size

Lesion size was measured as the longest in plane diameter, apart from lymph nodes or soft tissue mass (due to the difficulty in differentiating a soft tissue mass from a lymph node). In these lesions the short axis, i.e. the longest diameter perpendicular to the longest in plane diameter, was recorded as lesion size.

## Results

### Patient-based analysis

Seventeen of 28 patients (61%) showed complete concordance between the MRI and the PET/CT examinations. Eleven patients had positive findings on PET/CT. In these patients MRI showed at least one malignant lesion in 10 patients (91%). Thirteen patients had no discernible lesion with either modality. There were three patients with lesions that were detected by PET/CT but not with MRI (1–4 lesions per patient), four patients with liver lesions that were detected by MRI but not with PET/CT (1–7 lesions per patient), and three patients with MRI-only detected lesions in other locations (1 or 2 lesions per patient). One patient had 1 lesion that was only detected by PET/CT and 2 liver lesions that were only detected by MRI.

### Lesion-based analysis

**Figure 1.**
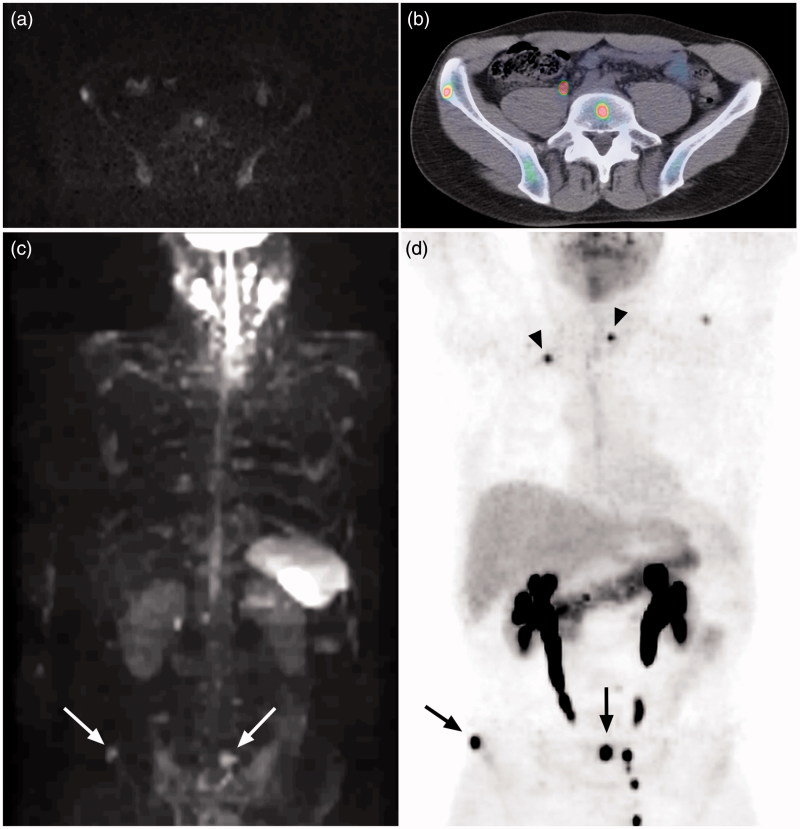
Bone marrow metastases in the lumbar spine and pelvis clearly visualized by both axial diffusion-weighted MRI (a) and 5-HTP PET/CT (b). The lesions in (a) and (b) are indicated (arrows) in MIP-images of diffusion-weighted MRI (c) and 5-HTP PET (d) in the same patient. The lymph node metastasis in thorax and bone marrow metastasis in vertebra Th II (arrow heads) are not detected with MRI (c), but clearly seen with PET (d). Hyperintense normal CNS, salivary glands, spleen, and lymph nodes are also seen in (c). Normal excretion of metabolites to the urinary tract is seen in (b) and (d).

A majority of the lesions were located in mediastinal lymph nodes, in the liver, or in the skeleton. Two patients had more than 10 lesions that were clustered together and impossible to distinguish from each other. Since the lesions could not be distinguished from each other, reliable comparisons between PET/CT and MRI for the individual lesions could not be performed. The patients have therefore been excluded from the lesion-based analysis (Table 5). These lesions were located in the mediastinum/upper thorax in one patient and in the skeleton in the other patient. In both these patients the lesions were visible on both PET/CT and MRI. In lesion-based analysis, 34 of the remaining 64 lesions (53% concordance) were detected by both MRI and PET/CT. Eight more lesions were detected by PET/CT only, and four of them were located in the thoracic lymph nodes. These thoracic lymph node metastases were all <1 cm in size. The median size was 0.6 cm, as compared with a median size of 1.5 cm of the lesions detected by both MRI and PET/CT in the same location. Twenty-two lesions were detected with MRI only. Eighteen of them were located in the liver and had a median size of 0.6 cm compared with a median size of 0.9 cm of the liver lesions detected by both modalities. There was only one of the bone marrow lesions that was not detected by MRI (Figure 1), and one was not detected by PET/CT.

**Table 5.** Number (*n*) and size (in cm) of lesions at different locations detected with both 5-HTP PET/CT and MRI (including diffusion-weighted and contrast-enhanced images) or with only one of the modalities.

	MRI and PET/CT		PET/CT only		MRI only		MRI	PET/CT
Site of lesion	*n*	Size (cm)^a^	*n*	Size (cm)^a^	*n*	Size (cm)^a^	Total *n*	Total *n*
Pancreas	1	3	2	1 (1)	1	2.2	2	3
Liver	17	0.9 (0.5–1.3)	0	–	18	0.6 (0.3–1.6)	35	17
Lung	0	–	1	1.7	0	–	0	1
Bone	8	1.3 (1.0–2.0)	1	0.9	1	1.3	9	9
Adnexa	1	2.2	0	–	0	–	1	1
Lymph nodes/soft tissue mass in abdomen	4	1.1 (1–1.3)	0	–	2	1.1 (1.0–1.2)	6	4
Thoracic lymph nodes	3	1.5 (1.1–1.8)	4	0.6 (0.5–0.7)	0	–	3	7

a Median (minimum–maximum).

When analyzing the lesions based on diagnosis (Table 6) most of the lesions only detected by PET/CT were found in patients with LCC, and most of the lesions only detected by MRI were found in patients with either P-NET or LCC.

**Table 6.** Number of lesions detected with both 5-HTP PET/CT and MRI (including diffusion-weighted and contrast-enhanced images) or with only one of the modalities in different types of NET.

				Total number	
Diagnosis	MRI and PET/CT	PET/CT only	MRI only	MRI	PET/CT
Si-NET	17	0	2	19	17
P-NET	5	2	12	17	7
LCC^a^	12	6	7	19	18
Thymic NET	>10	0	0	>10	>10
Ovarian NET	0	0	0	0	0
Unknown origin	0	0	1	1	0

a One LCC patient with >10 lesions in lymph nodes in mediastinum/upper thorax which were visible with both MRI and PET/CT is excluded from the table for clarity.

LCC: lung carcinoid; P-NET: pancreatic neuroendocrine tumor; Si-NET: small intestine neuroendocrine tumor.

## Discussion

We found that there was a patient-based concordance of 61% and a lesion-based concordance of 53% between the modalities. There was good concordance between MRI and PET/CT regarding metastases in the skeleton as well (Figure 1), which is in accordance with several previous studies that have shown whole-body MRI including DWI to be a valuable tool for detection of bone marrow metastases (14,15). A majority of lesions only detected by PET/CT were small and located in the thorax, predominantly in lymph nodes (Figure 1). Furthermore, MRI detected more liver lesions than 5-HTP PET/CT.

The discordant results between MRI and 5-HTP PET/CT might partly be accredited to the extreme sensitivity of 5-HTP PET/CT in detection of NET outside the liver, which enables it to detect very small lesions (3). In order to avoid interpretation of artifacts and normal lymph nodes as pathological findings in diffusion-weighted images, the cut-off for minimum lesion size was set at 1 cm. Both normal lymph nodes and metastatic lymph nodes may have a high signal intensity on DWI which makes them difficult to separate (5). Some studies have indicated that ADC values can be used to discriminate benign from malignant lymph nodes. There is, however, an overlap in the ADC values between malignant and benign nodes, and there is high observer variability in measuring the ADC value (16–21). Measuring of ADC values has not yet been proven to be a reliable tool in distinguishing benign from malignant lymph nodes. Moreover, in this study most lesions, lymph nodes as well as other lesions, were small (Table 5), rendering ADC measurements unreliable. Though lesion size is not a perfect criterion to discriminate benign lymph nodes from malignant, it is an established method used in the RECIST 1.1 criteria, where normal lymph nodes are defined as having a short axis <1 cm. In our study we found clearly positive lesions that were smaller than 1 cm on PET/CT images. All lymph node metastases not detected by MRI were smaller than 1 cm, indicating that the cut-off value might have been set to high; however, a lower cut-off value had most probably resulted in many false positive lesions. A majority of the lesions only detected by PET/CT were found in the lungs or mediastinum, areas where lesions are difficult to detect with DWI due to motion-caused signal loss (6), low proton density, as well as the effects of susceptibility (22). Only one bone marrow lesion was not detected by MRI. This lesion was located in vertebra Th II (Figure 1). The lower central mediastinum, including adjacent structures or the root of the neck, is among those areas of the body that are prone to imaging artifacts, which can obscure lesions (23).

**Figure 2.**
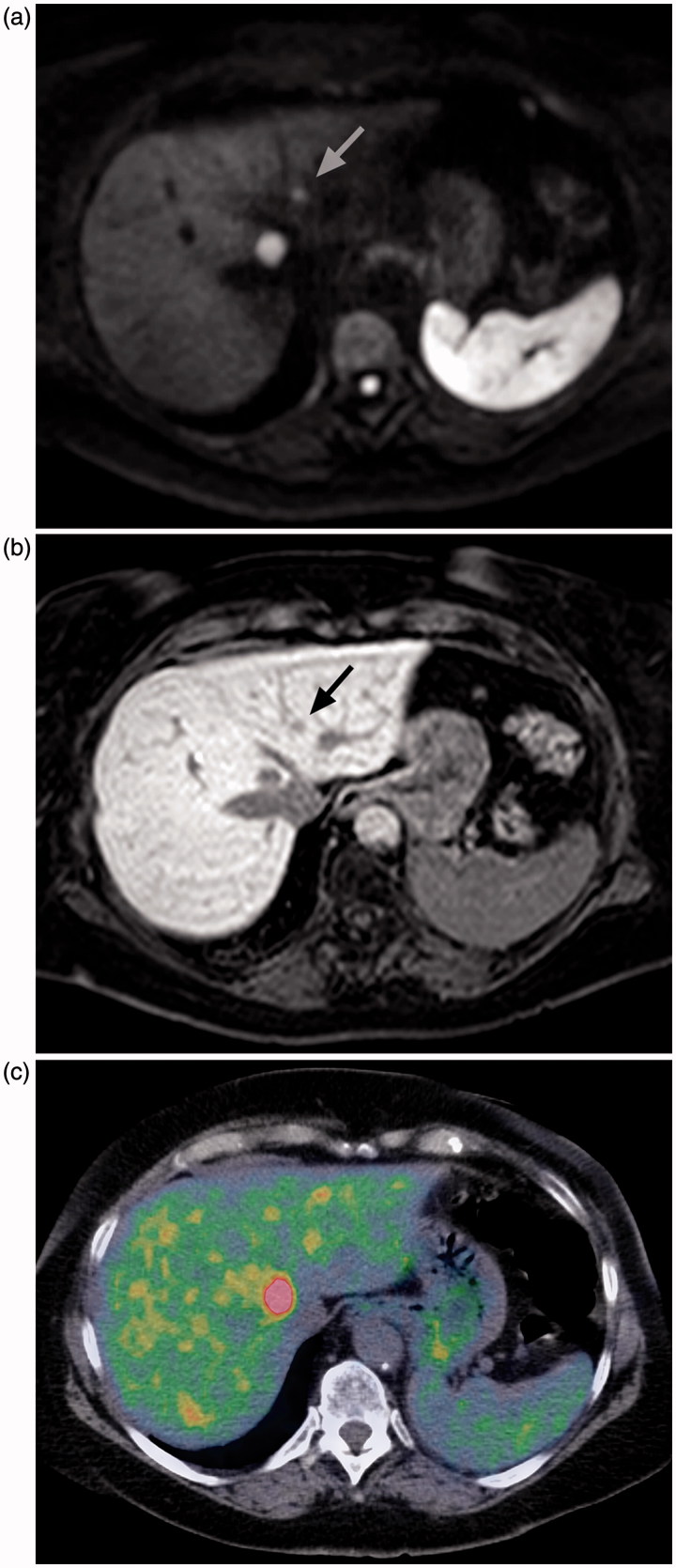
Axial diffusion-weighted MRI (a), contrast-enhanced MRI, hepato-biliary phase (b), and 5-HTP PET/CT (c) in the same patient. The larger lesion is clearly visible in all three, but the smaller one (indicated with arrow in MR images) is difficult to separate from the background uptake on the PET/CT image.

Seventeen liver lesions were found on PET/CT and 35 on MRI. The lesions found with MRI had a contrast-enhancement pattern typical of metastatic lesions, and several of them showed restricted diffusion, making it probable that they represent true lesions and not false positive. It has previously been shown that the combination of DWI and Gd-EOB-DTPA-enhanced imaging is of value in the detection of small liver metastases (24,25). The resolution of our PET camera is 0.5 cm and, as shown in Figure 2(C), there is a physiological background signal in the liver (13) due to the metabolism of serotonin (and thus the tracer) in the liver, making it difficult to detect liver lesions that are small or only have a moderate uptake of 5-HTP. That is probably the reason why 18 additional liver lesions were seen with MRI, since the median size of them was less than the median size of liver lesions detected with PET/CT. This interpretation of the findings is most clearly demonstrated by the patient who had four distinctly PET-positive lesions in the liver, i.e. metastases of NET. MRI of the same patient the following day showed seven additional smaller lesions. The smaller lesions exhibited the same pattern of contrast enhancement and restricted diffusion as the larger PET-positive lesions. That makes it highly probable that all the lesions were metastases of NET (Figure 2). This indicates that MRI of the liver could be a valuable complement to whole-body 5-HTP PET/CT. By combining 5-HTP PET with contrast-enhanced CT of thorax and abdomen more liver metastases would probably have been detected than by 5-HTP PET with low-dose CT alone. However, MRI with liver-specific contrast agent or DWI has been shown to be more sensitive for liver metastases than contrast-enhanced CT (26–28), and diagnostic contrast-enhanced CT entails a higher radiation dose.

Lesions only detected by MRI were mainly found in patients with P-NET or LCC (Table 6), and of these 19 lesions 16 were found in the liver. The lesions only detected by MRI found in patients with Si-NET were also located in the liver. Most of the lesions detected with PET/CT only were found in patients with LCC and constitute thoracic lesions, mainly in the lymph nodes, but one lesion was found in the lung and one in vertebra Th II. These findings indicate that whole-body MRI with DWI may be most advantageous in gastro-entero-pancreatic NET, since they mainly metastasize to the liver (29,30).

A recent study comparing ^68^Ga-DOTATATE PET/CT, SRSS SPECT/CT, and whole-body DWI in detection of NET indicated that PET/CT seems to be the most sensitive method (7). However, MRI had high accuracy and the advantage of no exposure to radiation. 5-HTP PET, as opposed to somatostatin receptor scintigraphy and ^68^Ga-DOTATOC/DOTATATE PET, has the ability to visualize NET independently of somatostatin receptor status, and has a significantly higher detection rate than somatostatin receptor scintigraphy and contrast-enhanced diagnostic CT (3). Unfortunately, no study comparing the detection rate of ^68^Ga-DOTATOC/DOTATATE and ^11^C-5-HTP has as yet been performed. One study showed that ^18^F-DOPA as a tracer in PET/CT was more sensitive than ^11^C-5-HTP in detecting Si-NET (sensitivity of 98% as compared to 89%, respectively), but ^11^C-5-HTP had a higher sensitivity in P-NET (96% compared to 80%) (31). However, we have previously shown a ^11^C-5-HTP sensitivity of up to 100% for detecting Si-NET (32). A recent study using ^68^Ga-DOTATOC as a tracer showed that PET/MR is equivalent to PET/CT in lesion detection of NET (33). The superior detection of liver metastases with MRI in our study strengthens the argument for exploring whole-body PET/MR as an option when using 5-HTP as a tracer.

This study included both treated and untreated patients. The fact that most patients had previously undergone or were undergoing treatment at the time of the imaging could explain why almost half of them had no discernible lesion with either modality. Patients with different types of NET were included, which may possibly cause bias due to tumor heterogeneity. It should also be taken into account that the patient material is rather small and the patients have been examined with different MR scanners and imaging protocols. Although all patients have been biopsied at the time of diagnosis, biopsy is not an option as standard of reference in this study, since biopsy of every single lesion detected would not be possible for ethical and logistical reasons. Using follow-up studies would not be a reliable method of distinguishing which lesions are true representatives of NET metastases, since many patients in this study had ongoing therapy. Therefore, PET/CT performed in close time correlation to the MRI study was the method that was considered as reference standard when interpreting the results for all locations apart from the liver (see above).

In conclusion, whole-body MRI including DWI and dedicated liver-specific contrast agent-enhanced imaging did not detect all NET lesions detected with whole-body 5-HTP PET/CT. However, the superior detection of liver metastases with liver-specific contrast agent MRI and DWI compared to 5-HTP PET/CT indicates that MRI of the liver could be a useful complement to whole-body 5-HTP PET/CT.
